# Multimorbidity and health seeking behaviours among older people in Myanmar: A community survey

**DOI:** 10.1371/journal.pone.0219543

**Published:** 2019-07-11

**Authors:** San Kyu Kyu Aye, Hlaing Hlaing Hlaing, San San Htay, Robert Cumming

**Affiliations:** 1 Department of Preventive and Social Medicine, University of Medicine Mandalay, Mandalay, Myanmar; 2 Department of Preventive and Social Medicine, University of Medicine 2, Yangon, Myanmar; 3 School of Public Health, Faculty of Medicine and Health, University of Sydney, Sydney, New South Wales, Australia; Ministry of Health and Sports, MYANMAR

## Abstract

**Background:**

The world population is aging very rapidly and the impact is more severe in developing countries because of insufficient resources and low awareness of the challenges faced by older people. This study aimed to explore multimorbidity of older people in Myanmar and their health seeking behaviours.

**Methods:**

A community-based cross-sectional study was conducted in both urban and rural areas of Bago Region and Mon State during October 2016. A multistage sampling method was used to select 4,859 people aged 60 years and older. Participants were interviewed face-to-face using a questionnaire. Multinominal logistic regression was used to analyse data.

**Results:**

More than half of the study participants (57.9%) reported at least one chronic condition in the last year and 33.2% reported two or more conditions (multimorbidity). The common conditions were hypertension (67.3%), arthritis (24.7%), arrhythmia (14.7%), coronary heart disease (13.8%) and diabetes (13.7%). A majority (61.7%) of participants with a chronic condition took western medicine. Older people usually saw a doctor (60.2%) or health assistant (21.9%) at a nearby clinic or rural health center; 1.6% reported seeing uncredentialed medical persons. Factors associated with multimorbidity were being female (adjusted Prevalence Ratio (aPR) = 2.14, 95% confidence interval (CI) 1.63–2.82) and having fair (aPR = 2.20, 95% CI 1.59–3.04) or poor self-reported health (aPR = 3.93, 95% CI 2.79–5.52). Those with less than middle school education (aPR = 0.50, 95% CI 0.25–0.99) and those living in rural areas (aPR = 0.78, 95% CI 0.62–0.98) were less likely to have multimorbidity. Older people in rural areas had less access to health care than their urban counterparts.

**Conclusion:**

Chronic conditions are common among older people in Myanmar, with higher prevalence in women and in urban areas. The lower prevalence of chronic conditions in those who live in rural areas may be related to living a more traditional lifestyle.

## Introduction

The world population is aging rapidly [1–3] and this is associated with an increasing occurrence of chronic diseases [4]. Older people tend to have more than one chronic condition, now known as multimorbidity [4–7]. Multimorbidity leads to poor quality of life, frequent hospitalization [4, 8], increased hospital visits [9], increased health care expenditure [4], higher stress, and worse health outcomes [10], including higher mortality [6]. Globally, the prevalence of multimorbidity among older people has been reported to range from 13% to 98% [4, 8, 11–16]. There is an increasing trend of multimorbidity in low- and middle-income countries (LMICs), due to epidemiological and demographic transitions, leading to changes in disease burden [17, 18] and an increase in high risk behaviours such as lack of physical exercise and smoking [11, 17].

The pattern of morbidity in developing countries is gradually becoming similar to developed countries [11]. The common morbidities among older people are hypertension, arthritis, diabetes mellitus, stroke, angina, cataract, cardiovascular disease, chronic lung disease, musculoskeletal disorder, ocular problems, urinary problems and sleeping problems. These conditions are frequent in both developed and developing countries, including Myanmar, neighbouring countries and other LMICs [5, 7, 8, 11–14, 18–22].

Knowing the patterns of multimorbidity in a specific community is crucial for management of people with multimorbidity [23] but the majority of evidence about multimorbidity in older people comes from high-income countries, with limited information from LMICs, including Myanmar [4, 11, 23, 24]. The current policy and practice of health care for older people in Myanmar is mainly focused on management of individual diseases leading to inefficient and ineffective health care [21].

In previous research in LMICs in Asia, multimorbidity has been found to be associated with increasing age, being female [8, 9, 12, 13], higher socio-economic status [9], higher education [12], unemployment, low income, and being an ex-drinker [5]. The most comprehensive study of health of older people is the World Health Organization (WHO) Study on global AGEing and adult health (SAGE), which was conducted in six LMICs: China, Ghana, India, Mexico, Russia and South Africa. SAGE found that multimorbidity was associated with increasing age, being female, and lower education level (except in China) and, in India, being separated/divorced/widowed [11]. A survey of people aged 18 and above in four Greater Mekong countries (Cambodia, Myanmar, Thailand, and Vietnam) found that multimorbidity was associated with older age, being male, lower education, and lower quality of life [16].

In Myanmar, the estimated total population was 51.4 million in the 2015 census [25] and about 9.1% were aged 60 and above [2, 21, 25]. The proportion of older people in Myanmar is projected to rise to 21.4% by 2050 [21, 26]. An increase in the older population is an emerging challenge for developing countries like Myanmar because of low awareness of the health problems of older people and limited health care services and social protection schemes for older people [1, 3, 21, 27, 28]. Furthermore, the traditional care pattern by children and relatives is now being challenged by a declining birth rate, urbanization and migration [2, 21, 28]. Another challenge is that the majority of the population lives in rural areas with limited access to care [2, 21]. This restricted access to healthcare is likely to lead to under-ascertainment and, hence, under-treatment of multimorbidity [4, 8, 29].

There have been previous studies in Myanmar of the health of older people [20, 27, 28, 30, 31], but to the best of our knowledge, there have been no previous studies of multimorbidity among older people. Therefore, this study aimed to describe the magnitude of multimorbidity and its risk factors, patterns of coexistence of diseases and health seeking behaviours among people aged 60 years and over in two areas of Myanmar (Bago Region and Mon State). Selecting these two areas was partly due to administrative and security concerns for data collectors (who were medical students). These two areas are representative of Myanmar in terms of socio-demographic characteristics and the combined older populations of Bago Region and Mon State represent 13.7% of older population in Myanmar [25].

## Methods

### Study design and setting

A community-based cross-sectional study was conducted during October, 2016, by 4^th^ year medical students of the University of Medicine, Mandalay in randomly selected townships in Bago Region and Mon State [25]. There are 15 states and regions in Myanmar and there were four medical universities when this study was done. A three-week residential field training program is required of all medical students, where they are assigned to particular townships for experience of medical and public health practice. For administrative and security reasons, we purposely excluded the states and regions that are hard to reach and have social instability, leaving six regions and three states which represent 84% of the population of Myanmar, from which we selected our study areas. Bago Region and Mon State were selected because they are representativeness of Myanmar’s socio-demographic characteristics. For example, the proportion of people aged 60 and over is 6.2% for Myanmar overall and 6.8% for the selected study areas; the rural:urban distribution is 70:30 for Myanmar overall and 74:26 for the study areas; and the old age dependency ratio is 8.8 in Myanmar overall and 9.8 in the study areas [25]. The study protocol was approved by the Ethical Review Committee of University of Medicine, Mandalay, Myanmar.

### Study sample and sampling procedure

Older people aged 60 years and above who had resided in the study areas for at least one year were included. There are 27 townships in Bago Region and 19 townships in Mon State and each township is composed of both urban wards and rural villages. The number of townships selected from each of the two areas reflected the proportion of older population in the combined population: 70.3% (438,063) in Bago Region and 29.7% (184,895) in Mon State [25].

A multistage sampling technique was used to select study subjects. Firstly, nineteen townships from Bago Region and eight townships from Mon State were selected by simple random sampling as the primary sampling units. All the townships selected from Bago Region and Mon State are similar in their socio-demographic characteristics such as the proportion of older people, the proportion of urban and rural residences, sex composition and social context [25]. For administrative reasons related to students’ (our data collectors) curriculum requirements, we selected three wards and three villages from each township. The secondary sampling units were three randomly selected wards in urban areas and three randomly selected villages in rural areas, giving a total of 81 wards and 81 villages. Finally, eligible people aged 60 and above were recruited from randomly selected households, the tertiary sampling unit. If there was more than one eligible participant in the selected household, one older person was selected to participate by a lottery method. If the eligible participant refused to take part in the interview after explanation of the study, the data collector noted this as a non-response. If the eligible older person was absent during the first data collection visit, the interviewer arranged to return at another time to do the interview. The participation rate was 99.9% of 4860 eligible participants.

### Data collection

After obtaining verbal informed consent, participants were interviewed face-to-face by trained 4^th^ year medical students using a semi-structured paper questionnaire. The questionnaire was developed in local language in which 14 chronic diseases or illnesses were included, based on government reports and findings from previous research in Myanmar and neighbouring countries [8, 12, 14, 16, 18, 20, 21, 32]. See supplementary document for more information (S1 File). The questionnaire was pretested and then modified in order to improve comprehension. Participants were asked about the occurrence of chronic diseases in the last 12 months: “During the last year, did you have any chronic condition or disease told to you by a doctor or other health persons such as nurse, health assistant, lady health visitor, or midwife?” If the older people responded “yes”, then the data collector asked “What is/are the chronic condition(s) or disease(s)?”, and they read out 14 listed chronic diseases: high blood pressure, coronary heart disease or heart attack, heart failure, irregular heart-beat, chronic bronchitis or chronic obstructive airway disease (COAD), asthma; stroke, diabetes, arthritis or rheumatoid arthritis, osteoporosis, glaucoma, cataract, depression, and emotional or mental illness. Participants could also report other diseases not included in the list. Participants were also asked about their health seeking behaviours when they were unwell: the usual place of seeking health care, the usual health care providers and the usual care givers.

Socio-demographic characteristics of participants were collected, including age, sex, ethnicity, marital status, types of family or composition of the family, level of education, types of occupation and monthly family income. Information on smoking, alcohol drinking and betel chewing was collected and these behaviours were grouped into current, never and ex-users. An older person who had ever smoked, drunk alcohol or chewed betel quid but had quit for at least 3 months was categorised as an ex-user.

General health status was recorded by participants rating their health as good, fair or poor. Eyesight and hearing status were recorded as good, fair, poor and completely blind or deaf. Self-assessed life satisfaction was recorded as satisfactory, fair and unsatisfactory. Further questions were asked about relationships with other family members, ability to do daily activities and involvement in social activities.

Quality control of the data was considered during data collection, entry and analysis phases. One day training on data collection was given to all medical students, including: introductory greetings and asking for verbal informed consent; non-directed prompting in asking questions; and checking the completeness and consistency of the data on the day of data collection. Data were collected by pairs of students, who both signed off on completed questionnaires.

### Data management and analysis

Data entry using Epidata software was done by the students themselves after returning back from their three-week community placement. Data were exported to Stata version 13 (Stata Corp, College Station, TX) for analysis. Data cleaning was conducted by the researchers together with the students. If there were unusual or missing data, cross-checking with the original paper questionnaire was done.

The primary outcome variable, number of chronic conditions, was categorized into no condition, one condition and two or more chronic conditions (multimorbidity). Sampling weights and the complex sampling design were taken into account during data analysis. Probability weights were calculated as the inverse of the sampling fraction for each stage (using the formula N/n, where N = the number of elements in the population and n = the number of elements in the sample). The probability weight for the whole data set was calculated by multiplication of the inverse of the sampling fraction for the first, second and third stages. We adjusted for the complex sampling design using “svyset” and performed data analysis applying the survey prefix command “svy” in Stata.

Socio-demographic and personal characteristics, self-assessed general health status, morbidity patterns, family support and life satisfaction are presented according to residence (urban or rural). Univariate and multinomial logistic regression models were used to identify factors associated with multimorbidity. Multinomial regression was used as the outcome had three levels: no morbidity, one morbidity and multimorbidity. Variables with a p value of <0.1 in univariate analysis were retained in the final multivariable model. P values <0.05 were considered to be statistically significant.

Exploratory factor analysis was performed by analyzing the clustering of 14 chronic diseases in order to derive multimorbidity patterns [33]. Minimum suggested thresholds of chronic disease prevalence for entering into exploratory factor analysis have ranged from 1–5% [23] and we considered 5% in our analysis. Sampling adequacy and inter-correlation of variables were checked using Bartlett’s test of sphericity and Kaiser-Meyer-Olkin’s measure. We used a tetrachoric correlation matrix which is an appropriate method for factor analysis of dichotomous data. The number of factors was determined based on the Kaiser criterion (eigenvalues >1) and scree plots. For easy interpretation of the factors, oblique rotation (Oblimin) was performed [34]. Morbidities with absolute factor loadings >0.3 were designated as a significant contributor to the derived morbidity pattern.

## Results

A total of 4,859 people aged 60 years and older participated in the study. Forty six percent were aged 70 and above (mean age and standard deviation 70 ± 8.2 years) and the age range was 60 to 106 years. Sixty two percent were female (male to female ratio 1:1.6) and 41.7% were divorced, separated or widowed. Only 12.5% of the older people lived in an extended family (three generation family plus first-degree relatives), 42.3% lived in a three-generation family and 45.2% lived in a nuclear two-generation family. Sixty eight percent had middle school or lower education and 65.1% were financially dependent on other family members. Twenty six percent lived in households having less than Kyats 100,000 family income per month (<3US$ per day) and 5.0% lived in a family with less than Kyats 35,000 per month (<1US$ per day). Regarding personal habits, 24.3% were current smokers, 4.7% were current drinkers and 33.7% were current betel chewers. Urban dwellers tended to have higher education and higher family income compared to their rural counterparts. In contrast, more older people in rural areas owned family businesses (33.2%) and were current smokers (29.8%) than those in urban areas (20.5% and 18.9%, respectively). All other characteristics were similar in urban and rural areas (Table 1).

**Table 1:** Socio-demographic and personal characteristics of study participants.

Characteristics	N	Urban	Rural	Total
**Age group (year)**	4859	N (%)	N (%)	N (%)
60–69		1330 (54.5)	1296 (53.6)	2626 (54.0)
≥ 70		1112 (45.5)	1121 (46.4)	2233 (46.0)
**Sex**	4859			
Male		879 (36.0)	962 (39.8)	1841 (37.9)
Female		1563 (64.0)	1455 (60.2)	3018 (62.1)
**Ethnicity**	4859			
Barma		1942 (79.5)	1915 (79.2)	3857 (79.4)
Other		500 (20.5)	502 (20.8)	1002 (20.6)
**Marital status**	4859			
Never married		175 (7.2)	176 (7.3)	351 (7.2)
Divorced/Separated/Widowed		1231 (50.4)	1251 (51.8)	2026 (41.7)
Married		1036 (42.4)	990 (41.0)	2482 (51.1)
**Type of family**	4859			
Nuclear		1050 (43.0)	1148 (47.5)	2198 (45.2)
Three generation		1050 (43.0)	1004 (41.5)	2054 (42.3)
Extended		342 (14.0)	265 (11.0)	607 (12.5)
**Level of education**	4859			
Illiterate		211 (8.6)	258 (10.7)	469 (9.7)
Primary to Middle school		1172 (48.0)	1670 (69.1)	2842 (58.5)
High school		846 (34.6)	448 (18.5)	1294 (26.6)
Diploma to Postgraduate		213 (8.7)	41 (1.7)	254 (5.2)
**Type of occupation**	4859			
Employee		105 (4.3)	83 (3.4)	188 (3.9)
Self-employed/Family business		500 (20.5)	802 (33.2)	1302 (26.8)
Dependent/ retired		1746 (71.5)	1416 (58.6)	3162 (65.1)
Others		91 (3.7)	116 (4.8)	207 (4.3)
Family Income (Kyats)**	4832			
≤ 35000		96 (3.9)	161 (6.7)	257 (5.3)
>35000–99999		403 (16.6)	593 (24.7)	996 (20.6)
100000–199999		821 (33.7)	862 (35.9)	1683 (34.8)
200000–299999		478 (19.6)	405 (16.9)	883 (18.3)
≥ 300000		637 (26.2)	376 (15.7)	1013 (21.0)
**Smoking status**	4859			
Current smoker		461 (18.9)	720 (29.8)	1181 (24.3)
Never smoker		1710 (70.0)	1467 (60.7)	3177 (65.4)
Ex-smoker		271 (11.1)	230 (9.5)	501 (10.3)
**Alcohol drinking status**	4859			
Current drinker		82 (3.4)	146 (6.0)	228 (4.7)
Never drinker		2201 (90.1)	2140 (88.5)	4341 (89.3)
Ex-drinker		159 (6.5)	131 (5.4)	290 (6.0)
**Betel chewing status**	4859			
Current betel chewer		762 (31.2)	877 (36.3)	1639 (33.7)
Never betel chewer		1569 (64.3)	1450 (60.0)	3019 (62.1)
Ex-betel chewer		111 (4.5)	90 (3.7)	201 (4.1)

*SD = Standard Deviation,

**1 USD = Kyats 1360 (Exchange rate at the time of interviews)

Regarding general health status, 27.9% rated their general health status as poor: 26.1% in urban areas and 29.8% in rural areas. More participants in both urban and rural areas rated their hearing as good than rated their eyesight as good. The majority (92.8% in urban and 91.3% in rural areas) were able to do daily activities by themselves and 36.1%were involved in community activities. Almost all respondents (95.8% in urban and 97.1% in rural areas) had a good relationship with their family. Concerning life satisfaction, 78.1% in urban and 75.3% in rural areas reported that they were satisfied with their lives (Table 2).

**Table 2:** General health status, relationship with the family and social involvement of study participants.

Characteristics	N	Urban	Rural	Total
**General Health Status**	4859	N (%)	N (%)	N (%)
Good		1107 (45.3)	1001 (41.4)	2108 (43.4)
Fair		698 (28.6)	697 (28.8)	1395 (28.7)
Poor		637 (26.1)	719 (29.8)	1356 (27.9)
**Eyesight**	4859			
Good		1102 (45.1)	1046 (43.3)	2148 (44.2)
Fair		622 (25.5)	627 (25.9)	1249 (25.7)
Poor		706 (28.9)	736 (30.5)	1442 (29.7)
Blind		12 (0.5)	8 (0.3)	20 (0.4)
**Hearing**	4859			
Good		1835 (75.1)	1752 (72.5)	3587 (73.8)
Fair		324 (13.3)	314 (13.0)	638 (13.1)
Poor		281 (11.5)	346 (14.3)	627 (12.9)
Deaf		2 (0.1)	5 (0.2)	7 (0.1)
**Ability to do daily activities**	4859			
Yes		2267 (92.8)	2207 (91.3)	4474 (92.1)
No		175 (7.2)	210 (8.7)	385 (7.9)
**Take part in social activities as volunteer**	4858			
Yes		901 (36.9)	854 (35.3)	1755 (36.1)
No		1540 (63.1)	1563 (64.7)	3103 (63.9)
**Good relationship with other family members**	4819			
Yes		2327 (95.8)	2320 (97.1)	4647 (96.4)
No		103 (4.2)	69 (2.9)	172 (3.6)
**Life satisfaction**	4859			
Satisfactory		1907 (78.1)	1821 (75.3)	3728 (76.7)
Fair		425 (17.4)	488 (20.2)	913 (18.8)
Unsatisfactory		110 (4.5)	108 (4.5)	218 (4.5)

**Fig 1:**
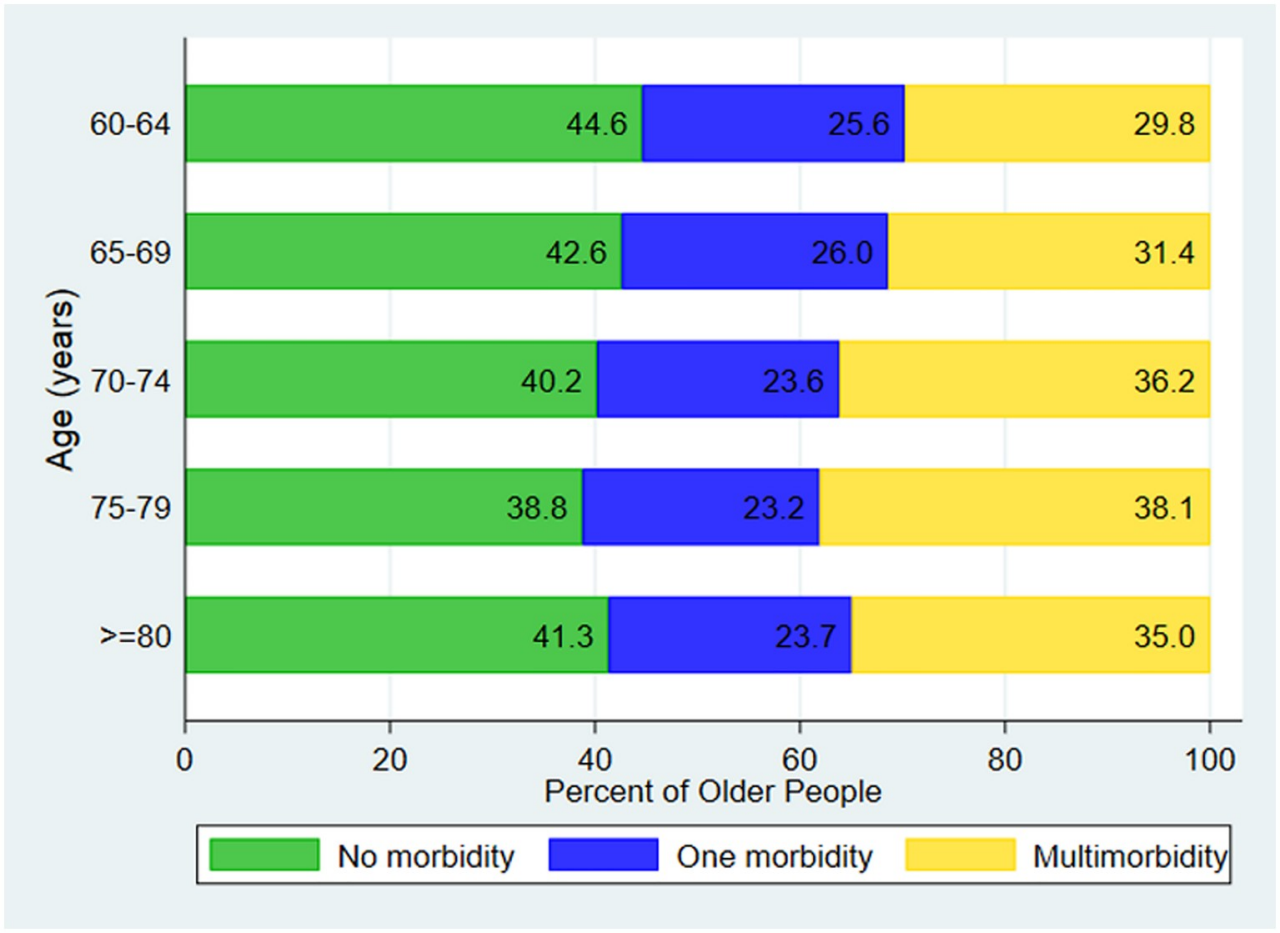
Morbidity patterns of older people by age group (n = 4859).

More than half of the participants [57.9% (95% CI 56.5–59.3%)] answered that they suffered from at least one chronic disease in the last year, 58.9% (95% CI 56.9–60.8%) in urban areas and 57.0% (95% CI 55.0–58.9%) in rural areas (Table 3). The prevalence of multimorbidity (two or more conditions) was 33.2% (95% CI 31.9–34.5%), increasing with age from 29.8% (95% CI 27.5–32.2%) in those 60–64 years to 38.1% (95% CI 34.5–41.7%) in those aged 75–79 years (Fig 1). The most common chronic conditions were high blood pressure (67.5%), arthritis (24.8%), arrhythmias (14.8%), coronary heart disease (13.8%), diabetes (13.8%) and cataract (13.0%). Among those who had chronic disease, 61.7% took western medicines regularly. The most common places for seeking care when unwell were general practitioner’s clinics (55.3%) in urban areas and Rural Health Centers (51.7%) in rural areas. Doctors (60.2%) followed by Health Assistants (21.9%) were the most commonly used health care providers. Most urban dwellers saw the doctors (79.8%) and an equal proportion of rural residences saw doctors (40.3%) and Health Assistants (39.2%). Only 1.6% reported being treated by untrained or uncredentialed medical personnel. The majority (96.8%) of the older people in our study reported that they had someone to look after them whenever they were sick, with 66.6% saying their children looked after them and 21.1% saying their spouse, with a similar pattern in both urban and rural areas (Table 3).

**Table 3:** Morbidity and health seeking behaviours of older study participants.

Characteristics	N	Urban	Rural	Total
**Suffering chronic disease last year**	4859	N (%)	N (%)	N (%)
No morbidity		1004 (41.1)	1040 (43.0)	2044 (42.1)
One morbidity		575 (23.6)	627 (26.0)	1202 (24.7)
Multimorbidity		863 (35.3)	750 (31.0)	1613 (33.2)
Common morbidities*	2815			
High Blood Pressure		942 (65.5)	952 (69.1)	1894 (67.3)
Arthritis		365 (25.4)	331 (24.0)	696 (24.7)
Arrhythmia		213 (14.8)	201 (14.6)	414 (14.7)
Coronary heart disease		238 (16.6)	150 (10.9)	388 (13.8)
Diabetes		237 (16.5)	149 (10.8)	386 (13.7)
Cataract		203 (14.1)	162 (11.8)	365 (13.0)
Others		922 (64.1)	797 (57.9)	1719 (61.1)
**Taking western medicine regularly**	2815			
Yes		938 (65.2)	798 (58.0)	1736 (61.7)
No		500 (34.8)	579 (42.1)	1079 (38.3)
Usual place of taking medical treatment*	4859			
GP clinic		1332 (54.6)	442 (18.3)	1774 (36.5)
Rural Health Center		143 (5.9)	1240 (51.3)	1383 (28.5)
Hospital		612 (25.1)	486 (20.1)	1098 (22.6)
Urban Health Center		198 (8.2)	162 (6.8)	360 (7.5)
Others		149 (6.2)	107 (4.5)	256 (5.3)
Usual seeking Health care providers*	4859			
Doctor		1949 (79.8)	975 (40.3)	2924 (60.2)
Health Assistant		112 (4.6)	946 (39.2)	1058 (21.9)
LHV/ MW		267 (11.0)	433 (18.0)	700 (14.5)
Untrained medical person		44 (1.8)	33 (1.4)	77 (1.6)
Others		81 (3.3)	69 (2.9)	150 (3.1)
**Having someone look after whenever got sick**	4859			
Yes		2359 (96.6)	2346 (97.1)	4705 (96.8)
No		83 (3.4)	71 (2.9)	154 (3.2)
Types of caregiver*	4859			
Daughters and sons		1560 (66.2)	1570 (67.0)	3130 (66.6)
Spouse		491 (20.8)	499 (21.3)	990 (21.1)
Relatives		311 (12.7)	279 (11.5)	590 (12.1)
Others		103 (4.2)	80 (3.3)	183 (3.8)

*Multiple responses, GP—General Practitioner, LHV—Lady Health Visitor, MW—Midwives

The exploratory factor analysis revealed five morbidity patterns which explained 47% of total variance. The five factors can be interpreted as “cardiovascular diseases” for factor 1, “stroke and psychological problems” for factor 2, “metabolic and respiratory disorders” for factor 3, “musculoskeletal disorders” for factor 4 and “ocular problems” for factor 5 (see Table 4).

**Table 4:** Loadings for the five-factor solution following an exploratory factor analysis based on tetrachoric correlation matrix.

	Factor 1	Factor 2	Factor 3	Factor 4	Factor 5
High blood pressure	0.071	0.022	**0.545**	-0.271	-0.172
Coronary heart disease or heart attack	**0.743**	-0.057	0.072	0.021	0.010
Heart failure	**0.723**	-0.001	-0.060	-0.023	-0.004
Irregular heart beat	**0.672**	0.070	-0.048	0.035	0.011
Chronic bronchitis or chronic obstructive airway disease	0.028	0.213	**-0.372**	0.047	-0.070
Asthma	0.068	0.078	**-0.657**	-0.135	-0.078
Stroke	0.009	0.276	**0.341**	-0.093	-0.020
Diabetes	0.002	0.134	**0.475**	0.104	0.027
Arthritis or rheumatoid arthritis	-0.034	0.016	-0.062	**0.761**	-0.043
Osteoporosis	0.081	0.019	0.105	**0.709**	0.007
Glaucoma	0.009	0.053	-0.001	-0.112	**0.749**
Cataract	0.005	-0.035	0.012	0.076	**0.738**
Depression	-0.001	**0.780**	0.001	0.052	-0.051
Emotional and mental illness	-0.002	**0.773**	-0.010	-0.026	0.072

Loadings are shown after the application of oblimin rotation: loadings are bold if they exceeded ± 0.3

Table 5 shows the results of multinomial logistic regression models to identify factors associated with a single chronic condition and with multimorbidity. Older people who lived in rural area had lower occurrence of multimorbidity than older people in urban areas [adjusted Prevalence Ratio (aPR = 0.78, 95% CI 0.62–0.98)]. The prevalence of multimorbidity was also lower in those with less than middle school education compared to those with a diploma or degree (aPR = 0.50, 95% CI 0.25–0.99). The prevalence was higher in females than males for both a single chronic condition (aPR = 1.46, 95% CI 1.16–1.85) and for multimorbidity (aPR = 2.14, 95% CI 1.63–2.82). As expected, multimorbidity was significantly more common in those with poor (aPR = 3.93, 95% CI 2.79–5.52) and fair self-rated health (aPR = 2.20, 95% CI 1.59–3.04) than in those with good self-rated health. Ex-smokers, but not current smokers, had higher prevalence of a single chronic condition than non-smokers (aPR = 1.59, 95% CI 1.02–2.46), and ex-drinkers had higher prevalence of multimorbidity than non-drinkers (aPR = 1.59, 95% CI 0.95–2.67) (Table 5). (S1 Table shows the prevalence, with 95% CIs, of no morbidity, one morbidity and multimorbidity according to characteristics of study participants.)

**Table 5:** Factors associated with having one morbidity and multimorbidity compared to no morbidity: Results of multinomial logistic regression analysis (n = 4796).

Characteristics	No morbidity (n = 2018) Vs One Morbidity (n = 1188)	No morbidity (n = 2018) Vs Multimorbidity (n = 1590)
	aPR* (95% CI)	aPR (95% CI)
**Residence**		
Urban	Reference	Reference
Rural	1.16 (0.91–1.47)	0.78 (0.62–0.98)
**Sex**		
Male	Reference	Reference
Female	1.46 (1.16–1.85)	2.14 (1.63–2.82)
**Level of Education**		
Diploma/ graduate	Reference	Reference
Middle to High school	0.59 (0.29–1.22)	0.60 (0.29–1.27)
Below Middle school	0.47 (0.22–0.98)	0.50 (0.25–0.99)
Illiterate	0.49 (0.23–1.05)	0.48 (0.22–1.05)
**Smoking status**		
Never smoker	Reference	Reference
Current smoker	1.18 (0.85–1.64)	0.87 (0.66–1.14)
Ex-smoker	1.59 (1.02–2.46)	1.32 (0.79–2.21)
**Alcohol drinking status**		
Never drinker	Reference	Reference
Current drinker	1.03 (0.66–1.61)	1.51 (0.83–2.72)
Ex-drinker	1.20 (0.67–2.15)	1.59 (0.95–2.67)
**General health status**		
Good	Reference	Reference
Fair	1.28 (0.95–1.74)	2.20 (1.59–3.04)
Poor	1.98 (1.40–2.81)	3.93 (2.79–5.52)
**Involved in social activities**		
Yes	Reference	Reference
No	1.14 (0.89–1.46)	1.24 (0.98–1.57)

*aPR = Adjusted Prevalence Ratio. PRs are adjusted for all other variables in the table.

## Discussion

Among older people in our study in Myanmar, 60% had at least one morbidity and 33% had multimorbidity. Older people living in rural areas and those with less education were less likely to report multimorbidity. Older women and those with worse self-reported health status were more likely to have multimorbidity. Older people living in rural areas had less access to health care than those living in urban areas, which may partly explain their lower levels of multimorbidity, as their chronic conditions may have gone undetected.

Similar prevalence of multimorbidity has been found in studies of older people in other LMICs, including India (31% in one study and 45% in another) [8, 9], Indonesia (36%) [13], and in the WHO SAGE Study in Ghana (48%), China (45%), and India (58%) [15]. A higher prevalence of multimorbidity was found in older people in other SAGE countries (63% in South Africa, 64% in Mexico, and 72% in Russia) and in European countries (68% in Finland, 69% in Poland and 69% in Spain) [11]. Much lower prevalence of multimorbidity was found in a study of older people in rural Vietnam (13%) [12]. A survey of people aged 18 and above in four Greater Mekong countries found a very high prevalence of multimorbidity of 73% [16], as did a study of older people living in rural China, where 90%had multimorbidity [14]. The probable explanation for the high prevalence of multimorbidity in these two studies is that they were conducted in health care settings—primary health care facilities in the Greater Mekong countries and hospitals in China.

Similarly high multimorbidity prevalences have been reported in high-income countries: 44% of Dutch and 37% of Spanish people aged 15 and above [24], 37% of British people aged 16–85 [35], 52% of Australians aged 40 and above [36], and 25% of Swiss and 51% of Hungarians aged 50 and above [37]. Overall, the prevalence of multimorbidity across sixteen European countries was reported to be 37% [37]. However, a higher prevalence was found in another study in Spain, with 67% of older people aged 65 having multimorbidity [38].

Variations in prevalence of multimorbidity might be due to different age compositions of study populations. For example, older people aged 60 and older were included in our study, while other studies have used lower age cu-points, including 50 years and older in SAGE and 18 years and older in the Greater Mekong study. Other methodological reasons for variations in multimorbidity prevalence across studies include differences in ascertainment of medical conditions (questionnaires, clinical examination or laboratory investigation) and differences in access to health care. There is no standard way of measuring multimorbidity. A review of multimorbidity indices found that the number of diseases included in multimorbidity indices ranged from 4 to 102, with a median of 14 chronic conditions [39]. In our study the most common morbidities were high blood pressure, arthritis, arrhythmias, coronary heart disease, diabetes and cataract. This is similar to the global pattern [11], and also similar to the nearby countries of Bangladesh, China, India, Indonesia and Vietnam, as well as other LMICs such as Brazil, Ghana, Mexico, Russia and South Africa, where the most common conditions are hypertension [8, 11–14, 19], cardiovascular disease, hypercholesterolemia [7, 12–14, 19], arthritis, gout, musculoskeletal problem [11–13, 19, 22], diabetes mellitus, respiratory disorders, ocular problems and sleeping problems [7, 8, 22].

Regarding multimorbidity patterns, “cardiovascular diseases”, “stroke and psychological problem”, “metabolic and respiratory disorders”, “musculoskeletal disorders”, and “ocular problems” were the most common clusters of chronic conditions in our study in Myanmar. Globally, the three common clusters of multimorbidity are “cardio-respiratory”, including angina, asthma, and chronic obstructive pulmonary disease, “metabolic diseases”, including diabetes, obesity, and hypertension, and “mental-articular disorders”, including arthritis and depression [11]. A review of two European countries found that cardiovascular and metabolic disorders, mental health problems and musculoskeletal diseases were the three most common clusters of morbidities [23]. A study in Brazil reported that cardio-metabolic, musculoskeletal-mental and respiratory disorders were the most common clusters of morbidities [19].

Our study found that older people who lived in rural areas had lower prevalence of multimorbidity. This could be due to under-diagnosis of chronic conditions because of limited access to health care. A study from India also reported that older persons in rural areas had lower prevalence of multimorbidity [9]. In contrast, significantly higher prevalence of multimorbidity was present in older rural populations in China, Ghana, and South Africa [11].

Older women in our study had higher prevalence of multimorbidity than men. This finding is comparable with most previous studies [8, 11–13]. In contrast, the Greater Mekong study found higher prevalence of multimorbidity in men, perhaps due to the younger age (age 18 and over) of its study population [16]. Older women in our study were more likely to be widowed or separated or divorced and unemployed than older men and this may have contributed to their poorer health status. Previous studies in the Greater Mekong countries and the six LMICs in the SAGE study found that being separated or divorced or widowed was related to multiple chronic conditions [11, 16]. We also found that older people with low education had lower prevalence of multimorbidity. This is the opposite to what has been found in previous studies in four Greater Mekong countries [16], in WHO SAGE countries (India, Russia and South Africa) and some European countries (Finland, Poland, Spain) [11] and in Brazil [19]. The education finding in our study might be explained by the fact that Myanmar is economically less developed than most other countries where multimorbidity has been studied. Less educated people in Myanmar may still be living a traditional lifestyle that protects against chronic disease.

Most people in our study received their health care from a government clinic. This is similar to Bangladesh, where a study found that most older people visited government hospitals and community clinics when they were unwell [22]. Similarly, in all six SAGE countries, most older people went to out-patient clinics and saw a doctor for their illnesses [15]. The usual health care providers in our study were medical doctors and Health Assistants but a small proportion were treated by untrained or uncredentialed medical personnel. The same pattern of health service utilization was found in a previous study in Myanmar [31]. Although older people in Myanmar usually seek medical care from trained health care providers, the use of untrained or uncredentialed medical personnel remains unacceptably high.

As in a previous study in Myanmar [28], we found that older people usually live in low-income households and depend on their family, which can lead to poor access to health care because out-of-pocket expenditure represents 81% of total health expenditure in Myanmar [20, 40]. Our study found that older rural residents had limited access to health care, with only 18% of older people in rural areas going to a clinic and only 40% seeing a doctor at all in the past year, compared to 55% of urban dwellers going to a clinic and 80% seeing a doctor.

In Myanmar, children and/or relatives looking after older people play a major role in aged care [2, 25] but this traditional care pattern is now being challenged by demographic transition [2, 26]. Informal care in developing countries for older people is provided in a setting of multigenerational households, unlike in richer countries where nearly all older people live separately from their adult children. The demographic transition, and associated modernization, will lead to a decline in the number of multigenerational households and a decline in the intensity of informal care that can be provided by families. The majority of the older people in our study were looked after by their children, spouse and relatives whenever they were ill, similar to previous reports from Myanmar [26, 41]. These findings indicate that the traditional norms and values in relation to older people are still intact in Myanmar society. In the future, however, a decreasing fertility rate and migration of young adults are likely to decrease the availability of support from family members, resulting in the need for alternative approaches to care [2, 28].

A strength of our study is that we conducted a large population-based study using random sampling methods with a very high participation rate. By design, we included an equal proportion of urban and rural older people making findings generalizable to both urban and rural population. However, our study also has several limitations. We collected all data by questionnaire and so we relied on accurate reporting by study subjects. Data were collected by a large number of 4^th^ year medical students and so inter-observer variation is likely; however, all interviewers were trained in order to reduce this variation as much as possible. Our study findings may not be generalizable to older people living in social unstable areas, hard to reach areas and the large cities of Yangon and Mandalay. Another possible limitation of our study is that under-reporting of diseases might be a problem in LMICs like Myanmar with limited access to health care [11], leading to underestimation of the magnitude of the problem of chronic disease and multimorbidity.

### Implication for further research, health policy and services

Our study provides information that can be used to develop policies and plans for health care services to address the complex health needs of older people in Myanmar. To overcome the problem of underdiagnosis and, hence, under-reporting of chronic disease, future surveys should include objective measures such as blood pressure, blood sugar and serum cholesterol levels. More research is needed to provide evidence for deciding on the best package of interventions for older people in Myanmar, including screening programs, volunteer home-based care programs, programs targeting people in rural areas and long-term care for those who live alone. The government should also develop strategies to facilitate community programs to increase older people’s participation in social activities; income generation for older people who are able to work; and promotion of the traditional approach to caring for older people.

## Supporting information

S1 File Questionnaire (English and Burmese). (PDF)

S1 Table The percentage with 95% confidence intervals of no morbidity, one morbidity and multimorbidity by selected characteristics of study participants. (DOCX)

S1 Data Data set. (XLS)
