# Supplementary material for: Multimorbidity and health seeking behaviours among older people in Myanmar: A community survey
Source: PLoS One. 2019 Jul 11;14(7):e0219543. doi: 10.1371/journal.pone.0219543 (PMC6622547; doi:10.1371/journal.pone.0219543)
Supplement: S1 File — (PDF) [file pone.0219543.s001.pdf]

S1A File. Questionnaire (English)

University of Medicine, Mandalay

Department of Preventive and Social Medicine

Questionnaire for assessing morbidity and health seeking behaviour of older people aged 60 and above

This questionnaire is for assessing morbidity among older people and their health seeking behaviour. We would like to invite you to take part in this study. It is only for research purpose and your response will be confidentially kept.

Date………. Township…………. ☐☐ Ward/Village………. ☐☐ Serial number ☐☐☐☐

I. Questions relating personal characteristics of the respondents

SN	Questions	Code
P1	Age …………years (completed)	
P2	Gender (1) Male (2) Female	
P3	Marital status<br>(1) Never married (2) Currently married<br>(3) Divorced (4) Separated<br>(5) Widow/er	
P4	Type of family<br>(1) Nuclear Family (Father, mother and children)<br>(2) Three Generation Family (Grandparents, Father, mother and children)<br>(3) Extended Family (Grandparents, parents, children and other relatives)	
P5	Education level of the respondent<br>(1) Illiterate (2) Can read and write (No formal schooling)<br>(3) Primary school level (1-4 standard) (4) Middle school level (5-8 standard)<br>(5) High school level (9-10 standard) (6) Diploma<br>(7) University level (8) Graduate/ Post-graduate	
P6	Occupation of the respondent<br>(1) Government staff (2) Own business<br>(3) Involve in family business (4) Company staff/ worker<br>(5) Dependent (6) Retired<br>(7) Others (specify) ………	
P7	Average monthly family income (kyats)…………………………………….	
P8	Ethnicity of the respondent<br>(1) Bamar (2) Ethnic tribes…… (3) Others (specify)………..	
P9	Have you ever smoked?<br>(1) Yes (2) No (3) Ex-smoker (≥3 months)	
P10	Have you ever drunk alcohol?<br>(1) Yes (2) No (3) Ex-drinker (≥3 months)	
P11	Have you ever chewed betel quids?<br>(1) Yes (2) No (3) Ex-betel chewer (≥3 months)	

II. Questions asking about chronic conditions or diseases of the older people within the previous 12 months

SN	Questions	Code
1	**Currently,** how do you want to rate your general health status?<br>(1) Good (2) Fair (3) Poor	
2	**Currently,** how do you want to rate your eyesight status? (for those who wear glasses, consider current glasses wearing condition)<br>(1) Good (2) Fair (3) Poor (4) Completely Blind	
3	**Currently,** how do you want to rate your hearing status?<br>(1) Good (2) Fair (3) Poor (4) Deaf	
4	**During the last year,** did you have any chronic condition or disease told by doctor and other health persons such as nurse, health assistant, lady health visitor, and midwife?<br>(1) Yes (2) No (skip to Question 6)	
5	**If YES**, what is /are the chronic condition(s) or disease(s)? (Please read the list and also ask “Do you have other diseases that did not mention in the list?”)<br>(1) High Blood Pressure (1) Yes (2) No<br>(2) Coronary heart disease or Heart attack (1) Yes (2) No<br>(3) Heart Failure (1) Yes (2) No<br>(4) Irregular heart beat (1) Yes (2) No<br>(5) Chronic bronchitis or COAD (1) Yes (2) No<br>(6) Asthma (1) Yes (2) No<br>(7) Stroke (1) Yes (2) No<br>(8) Diabetes (1) Yes (2) No<br>(9) Arthritis or Rheumatoid arthritis (1) Yes (2) No<br>(10) Osteoporosis (1) Yes (2) No<br>(11) Glaucoma (1) Yes (2) No<br>(12) Cataract (1) Yes (2) No<br>(13) Depression (1) Yes (2) No<br>(14) Emotional & mental illness (1) Yes (2) No<br>(15) Other (specify)………………………..	
6	Have you ever regularly taken western medicine to control above mentioned chronic condition(s) or disease(s)?<br>(1) Yes (2) No	
7	Where do you usually go to seek health care when you feel ill/sick? (Multiple responses)<br>(1) Government hospital/clinic (2) Urban health centre<br>(3) Rural health centre (4) Nearby GP clinic<br>(5) Traditional medicine clinic (6) Other (specify)……..	

SN	Questions	Code
8	Who is/are the usual health care provider(s) when you got sick/ill? (Multiple responses)<br>(1) Doctor (2) Health assistant<br>(3) Lady health visitor/ midwife (4) Public health supervisor (I/II)<br>(5) Traditional medical personnel (6) Untrained medical personnel<br>(7) Other (specify)…………….	
9	Do you have anyone else to look after when you feel sick?<br>(1) Yes (2) No (skip to Question 11)	
10	If you have, please mention it. (Multiple responses)<br>(1) Spouse (2) Son/daughter<br>(3) Nephew/ niece (4) Relatives<br>(5) Neighbours (6) Other (specify)……………….	
11	Can you do your daily activity without help from anyone else?<br>(1) Yes (2) No	
12	Do you have a good relationship with other family members?<br>(1) Yes (2) No	
13	Do you take part in any social/ welfare activity?<br>(1) Yes (2) No	
14	Do you satisfy your current life condition?<br>(1) Satisfactory (2) Fair (3) Unsatisfactory	

Interviewer:

Student name ______________________ Roll number ____________ Signature _____

Counter check by

Student name ______________________ Roll number ____________ Signature _____

S1B File. Questionnaire (Burmese)

ဆေးတက္ကသိုလ်-မန္တလေး

ကာကွယ်ရေးနှင့်လူမှုရေးဆေးပညာဌာန

သက်ကြီးရွယ်အိုများ၏ နာမကျန်းဖြစ်မှု၊ ကျန်းမာရေးစောင့်ရှောက်မှုနှင့်ပက်သက်၍ ဆန်းစစ်သောမေးခွန်းလွှာ

ဤဆန်းစစ်လွှာသည် အသက် ၆၀နှစ် နှင့် အထက် သက်ကြီးရွယ်အိုများ၏ နာမကျန်းဖြစ်မှု၊ ကျန်းမာရေးစောင့်ရှောက်မှုနှင့် ပက်သက်၍ ဆန်းစစ်သော မေးခွန်းလွှာ ဖြစ်ပါသည်။ သုတေသနလုပ်ငန်းတွင် ပါဝင်ရန်အတွက် အဖိုး (သို့မဟုတ်) အဖွား ကို ဖိတ်ခေါ်အပ်ပါသည်။ ဤမေးမြန်းခြင်းသည် သုတေသနပြုလုပ်ရာတွင် အထောက်အကူပြုစေရန်အတွက် မေးမြန်းခြင်းသာ ဖြစ်ပြီး အဖိုး (သို့မဟုတ်) အဖွား ၏ ဖြေကြားချက်များကို လျှို့ဝှက်ပေးထားမည် ဖြစ်ပါသည်။

နေ့စွဲ ____________ မြို့နယ် ____________ ☐☐ ရပ်ကွက်/ရွာ ____________ ☐ အမှတ် ☐☐☐☐

(က) <u>ဖြေဆိုသူ၏ကိုယ်ရေးအချက်အလက်ဆိုင်ရာမေးခွန်းများ</u>

စဉ်	မေးခွန်းများ	Code
P1	ဖြေဆိုသူ၏ပြည့်ပြီးအသက် ________ နှစ်	
P2	ကျား/မ (၁) ကျား (၂) မ	
P3	ဖြေဆိုသူ၏အိမ်ထောင်ရေး<br>(၁) တစ်ကြိမ်မှအိမ်ထောင်မပြုဖူးသူ (၂) လက်ရှိအိမ်ထောင်ရှိ<br>(၃) တရားဝင်ကွဲ (၄) တရားဝင်မကွဲသေးအတူမနေ<br>(၅) မုဆိုးဖို/မုဆိုးမ	
P4	မိသားစုအမျိုးအစား<br>(၁) Nuclear Family (အဖေ၊အမေ၊သား-သမီးပါဝင်သောမိသားစု)<br>(၂) Three Generation Family (အဖိုး၊အဖွား၊အဖေ၊အမေ၊သား-သမီးပါဝင်သောမိသားစု)<br>(၃) Extended Family (အဖိုး၊အဖွား၊အဖေ၊အမေ၊သား-သမီးနှင့်အခြားဆွေမျိုးတော်စပ်သူများ ပါဝင်သောမိသားစု)	
P5	ဖြေဆိုသူ၏ပညာအရည်အချင်း (အောင်ပြီးအတန်း)<br>(၁) စာမတတ် (၂) ရေးတတ်ဖတ်တတ်/ဘုန်းကြီးကျောင်းထွက်<br>(၃) မူလတန်း (သူငယ်တန်းမှ၄တန်းထိ) (၄) အလယ်တန်း (၅တန်းမှ၈တန်းထိ)<br>(၅) အထက်တန်း (၉တန်းမှ၁၀တန်းထိ) (၆) ဒီပလိုမာ<br>(၇) တက္ကသိုလ်/ကောလိပ် (၈) ဘွဲ့ရ/ ဘွဲ့လွန်နှင့်အထက်	
P6	ဖြေဆိုသူ၏အလုပ်အကိုင်<br>(၁) အစိုးရဝန်ထမ်း (၂) ကိုယ်ပိုင်လုပ်ငန်း<br>(၃) မိသားစုလုပ်ငန်းတွင်လုပ်ကိုင်သူ (၄) အလုပ်သမား (ပုဂ္ဂလိက/အဖွဲ့အစည်း)<br>(၅) မှီခို (၆) အငြိမ်းစား<br>(၇) အခြား (တိတိကျကျဖော်ပြရန်) ____________________	
P7	သင့်မိသားစု၏ တစ်လ ပျမ်းမျှ ဝင်ငွေ ________________ ကျပ် (စာသားဖြင့်) ________________ ကျပ်	
P8	လူမျိုး (၁) ဗမာ (၂) တိုင်းရင်းသား ____________ (၃) အခြား (တိတိကျကျ ဖော်ပြရန်)__________	
P9	ဆေးလိပ်သောက်ပါသလား။<br>(၁) သောက်ပါသည်။ (၂) မသောက်ပါ။ (၃) ဆေးလိပ်ဖြတ်ထားပါသည်။ (၃လနှင့်အထက်)	
P10	အရက်သောက်ပါသလား။<br>(၁) သောက်ပါသည်။ (၂) မသောက်ပါ။ (၃) အရက်ဖြတ်ထားပါသည်။ (၃လနှင့်အထက်)	
P11	ကွမ်းစားပါသလား။<br>(၁) စားပါသည်။ (၂) မစားပါ။ (၃) ကွမ်းဖြတ်ထားပါသည်။ (၃လနှင့်အထက်)	

(ခ) အသက်(၆၀)နှစ်နှင့်အထက် သက်ကြီးရွယ်အိုများ၏ တစ်နှစ်အတွင်း နာမကျန်းမှုနှင့်ပက်သက်သော မေးခွန်းများ

စဉ်	မေးခွန်းများ	Code
1	**ယခုလတ်တလော**၊ သင်၏ကျန်းမာရေးအခြေအနေ (General Health) ကို မည်ကဲ့သို့ သတ်မှတ်လိုပါသလဲ။<br>(၁) ကောင်းပါသည်။ (Good) (၂) အသင့်အတင့်ကောင်းပါသည်။ (Fair)<br>(၃) မကောင်းပါ။ (Poor)	
2	**ယခုလတ်တလော**၊ သင်၏အမြင်အာရုံမည်သို့ရှိပါသဘဲ။ (မျက်မှန်၊မျက်ကပ်မှန်တပ်သူများအတွက် ၎င်းအရာများကို တပ်ဆင်ထားသည့်အခြေအနေ)<br>(၁) ကောင်းပါသည်။ (Good) (၂) အသင့်အတင့်ကောင်းပါသည်။ (Fair)<br>(၃) မကောင်းပါ။ (Poor) (၄) မမြင်ရပါ။ (Completely Blind)	
3	**ယခုလတ်တလော**၊သင်၏အကြားအာရုံမည်သို့ရှိပါသလဲ။<br>(၁) ကောင်းပါသည်။ (Good) (၂) အသင့်အတင့်ကောင်းပါသည်။ (Fair)<br>(၃) မကောင်းပါ။ (Poor) (၄) မကြားရပါ။ (Deaf)	
4	**လွန်ခဲ့သောတစ်နှစ်အတွင်း** ဆရာဝန်နှင့် အခြားကျန်းမာရေးဝန်ထမ်းများ (သူနာပြု၊ ကျန်းမာရေးမှူး၊ အမျိုးသမီး ကျန်းမာရေး ဆရာမ၊ သားဖွားဆရာမ)က သင့်မှာ နာတာရှည် ရောဂါတစ်မျိုးမျိုး ရှိတယ်လို့ ပြောဖူးပါသလား။<br>(၁) ရှိပါသည်။ (၂) မရှိပါ။ (မေးခွန်း ၆ သို့ ကျော်မေးပါ။)	
5	**(ရှိခဲ့ပါက)** အောက်ဖော်ပြပါ ရောဂါအမည် စာရင်းကို ဖတ်ပြပါ။ ထို့နောက် "သင့်မှာ ဤရောဂါများ အပြင် အခြား နာတာရှည်ရောဂါများ ရှိပါသေးသလား "ဟု မေး၍ ရှိပါက ရောဂါအမည်ကို ရေးမှတ် ထားရန်)<br>(၁) သွေးတိုး (High BP) ၁။ ရှိသည် ၂။ မရှိပါ။<br>(၂) နှလုံးသွေးကြောကျဉ်း (Coronary heart disease or Heart attack) ၁။ ရှိသည်။ ၂။ မရှိပါ။<br>(၃) နှလုံးအမောဖောက်၍ဆေးရုံတက်ရခြင်း (Heart Failure) ၁။ ရှိသည်။ ၂။ မရှိပါ။<br>(၄) နှလုံးခုန်နှုန်းမမှန်ခြင်း (Irregular heart beat) ၁။ ရှိသည် ၂။ မရှိပါ။<br>(၅) နာတာရှည်လေပြွန်ရောင်ခြင်း (Chronic bronchitis or COAD) ၁။ ရှိသည် ၂။ မရှိပါ။<br>(၆) ပန်းနာရင်ကြပ် (Asthma) ၁။ ရှိသည် ၂။ မရှိပါ။<br>(၇) လေဖြတ်ခြင်း/လေဖျန်းခြင်း (Stroke) ၁။ ရှိသည် ၂။ မရှိပါ။<br>(၈) ဆီးချိုသွေးချို (Diabetes) ၁။ ရှိသည် ၂။ မရှိပါ။<br>(၉) အဆစ်မြစ်ရောင်ရမ်းခြင်း (Arthritis or Rheumatoid arthritis) ၁။ ရှိသည် ၂။ မရှိပါ။<br>(၁၀) အရိုးပွခြင်း (Osteoporosis) ၁။ ရှိသည် ၂။ မရှိပါ။<br>(၁၁) ရေတိမ် (Glaucoma) ၁။ ရှိသည် ၂။ မရှိပါ။<br>(၁၂) အတွင်းတိမ် (Cataract) ၁။ ရှိသည် ၂။ မရှိပါ။<br>(၁၃) စိတ်ကျရောဂါ (Depression) ၁။ ရှိသည် ၂။ မရှိပါ။<br>(၁၄) အခြားသောစိတ်ရောဂါများ (Emotional & mental illness) ၁။ ရှိသည် ၂။ မရှိပါ။<br>(၁၅) အခြား (တိတိကျကျဖော်ပြပါ။) ____________________	
6	အထက်တွင် ဖော်ပြခဲ့သည့်နာတာရှည် ရောဂါကို ကုသရန်အတွက် အနောက်တိုင်းဆေး/အင်္ဂလိပ်ဆေး ကို ပုံမှန်သောက်သုံးနေရခြင်း ရှိပါသလား။<br>(၁) ရှိပါသည်။ (၂) မရှိပါ။	
7	နေမကောင်းဖြစ်ပါက ကျန်းမာရေးစောင့်ရှောက်မှုရယူရန် မည်သည့်နေရာသို့ သွားလေ့ရှိပါသလဲ။ **(တစ်မျိုးထက်မက ဖြေဆိုနိုင်ပါသည်။)**<br>(၁) အစိုးရဆေးရုံ/ ဆေးခန်း (၂) ဒေသန္တရဆေးခန်း<br>(၃) ကျေးလက်ကျန်းမာရေးဌာန (၄) နီးစပ်ရာပြင်ပဆေးခန်း<br>(၅) တိုင်းရင်းဆေးခန်း (၆) အခြား(တိတိကျကျဖော်ပြပါ) ____________	

စဉ်	မေးခွန်းများ	Code
8	ကျန်းမာရေးစောင့်ရှောက်မှုပေးသောသူမှာ **(တစ်မျိုးထက်မက ဖြေဆိုနိုင်ပါသည်။)**<br>(၁) ဆရာဝန် (၂) ကျန်းမာရေးမှူး<br>(၃) အမျိုးသမီးကျန်းမာရေးဆရာမ/သားဖွားဆရာမ (၄) ကျန်းမာရေးကြီးကြပ်(၁/၂)<br>(၅) တိုင်းရင်းဆေးဆရာ (၆) ရမ်းကု / စနစ်တကျသင်ယူတတ်မြောက်ထားခြင်းမရှိဘဲ ဆေးဝါးကုသနေသူ<br>(၇) အခြား(တိတိကျကျဖော်ပြပါ)______________________	
9	နေမကောင်းဖြစ်သောအခါ စောင့်ရှောက်ပေးမည့်သူ ရှိပါသလား။<br>(၁) ရှိပါသည်။ (၂) မရှိပါ။ (မေးခွန်း ၁၁ သို့ ကျော်မေးပါ။)	
10	ရှိပါက မည်သူဖြစ်ပါသလဲ။ **(တစ်မျိုးထက်မက ဖြေဆိုနိုင်ပါသည်။)**<br>(၁) အိမ်ထောင်ဖက် (၂) သား/သမီး<br>(၃) တူ/တူမ (၄) ဆွေမျိုးသားချင်း<br>(၅) အိမ်နီးနားချင်း (၆) အခြား (တိတိကျကျဖော်ပြပါ)______________________	
11	ပုံမှန်ပြုလုပ်နေကျ နေ့စဉ်လုပ်ငန်းများကို အကူအညီမပါဘဲ လုပ်နိုင်ပါ သလား။<br>(၁) လုပ်နိုင်ပါသည်။ (၂) မလုပ်နိုင်ပါ။	
12	အိမ်သားများနှင့် ဆက်ဆံရေး အခြေအနေ အဆင်ပြေမှု ရှိပါသလား။<br>(၁) ရှိသည် (၂) မရှိပါ။	
13	လူမှုရေး/ပရဟိတ လုပ်ငန်းများတွင် ပါဝင်ဆောင်ရွက်မှု ရှိပါသလား။<br>(၁) ရှိပါသည်။ (၂) မရှိပါ။	
14	မိမိလက်ရှိ နေထိုင်နေရသော ဘဝကို ကျေနပ်မှုရှိပါသလား။<br>(၁) ကျေနပ်ပါသည်။ (၂) အသင့်အတင့်ကျေနပ်ပါသည်။ (၃) မကျေနပ်ပါ။	

မေးမြန်းသော ကျောင်းသားအမည် ____________________ ခုံအမှတ် ___________ လက်မှတ် _________

ပြန်လည်စစ်ဆေးသော ကျောင်းသားအမည် _____________ ခုံအမှတ် ___________ လက်မှတ် ___________
