# Supplementary material for: Multimorbidity and health seeking behaviours among older people in Myanmar: A community survey
Source: PLoS One. 2019 Jul 11;14(7):e0219543. doi: 10.1371/journal.pone.0219543 (PMC6622547; doi:10.1371/journal.pone.0219543)
Supplement: S1 Table — (DOCX) [file pone.0219543.s002.docx]

S1 Table. Percentages with 95% confidence intervals of no morbidity, one morbidity and multimorbidity by selected characteristics of study participants

| Characteristics | No Morbidity  (n=2018) | One Morbidity  (n=1188) | Multimorbidity  (n=1590) |
| --- | --- | --- | --- |
|  | % (95%CI) | % (95%CI) | % (95%CI) |
| Residence |  |  |  |
| Urban | 49.4 (47.2 – 51.6) | 48.0 (45.1 – 50.8) | 53.8 (51.3 – 56.2) |
| Rural | 50.6 (48.4 – 52.8) | 52.0 (49.2 – 54.9) | 46.2 (43.8 -48.7) |
| Sex |  |  |  |
| Male | 44.3 (42.1 – 46.5) | 37.2 (34.4 – 40.0) | 30.0 (27.9 – 32.4) |
| Female | 55.7 (53.5 – 57.9) | 62.8 (60.0 -65.5) | 70.0 (67.6 – 72.1) |
| Level of Education |  |  |  |
| Diploma/ graduate | 5.1 (4.2 – 6.1 ) | 5.6 (4.5 – 7.1) | 5.3 (4.3 – 6.6) |
| Middle to High school | 29.1 (27.1 – 31.1) | 25.4 (23.0 – 28.0) | 24.8 (22.8 – 27.0) |
| Below Middle school | 56.4 (54.3 – 58.6) | 60.0 (57.0 – 62.6) | 60.0 (57.4 – 62.3) |
| Illiterate | 9.4 (8.2 – 10.8) | 9.1 (7.6 – 10.9) | 10.0 (8.6 – 11.5) |
| Smoking status |  |  |  |
| Never smoker | 64.4 (62.3 – 66.4) | 63.9 (61.1 – 66.6) | 68.1 (65.7 – 70.3) |
| Current smoker | 26.4 (24.5 – 28.4) | 26.8 (24.3 -29.3) | 19.5 (17.6 – 21.5) |
| Ex-smoker | 9.2 (8.0 – 10.5) | 9.3 (7.8 – 11.1) | 12.5 (10.9 -14.2) |
| Alcohol drinking status |  |  |  |
| Never drinker | 88.7 (87.2 – 90.0) | 89.2 (87.3 – 90.9) | 90.4 (88.9 – 91.8) |
| Current drinker | 5.7 (4.8 – 6.8) | 4.8 (3.7 – 6.2) | 3.1 (2.4 – 4.1) |
| Ex-drinker | 5.7 (4.7 – 6.7 ) | 6.0 (4.8 – 7.5) | 6.4 (5.3 – 7.7) |
| General health status |  |  |  |
| Good | 54.2 (52.0 – 56.4) | 45.1 (42.3 – 48.0) | 28.4 (26.2 – 30.7) |
| Fair | 27.1 (25.1 – 29.0) | 28.6 (26.1 – 31.2) | 30.9 (28.7 – 33.2) |
| Poor | 18.7 (17.1 – 20.5) | 26.2 (23.8 – 28.9) | 40.7 (38.3 – 43.1 ) |
| Involved in social activities | | | |
| Yes | 40.6 (38.4 – 42.7) | 35.4 (32.8 – 38.2) | 31.2 (29.0 – 33.5) |
| No | 59.4 (57.3 – 61.5) | 64.6 (61.8 – 67.2 ) | 68.8 (66.5 – 71.0) |
